# Comparable Clinical Outcome Using Small or Large Gross Tumor Volume-to-Clinical Target Volume Margin Expansion in Neoadjuvant Chemoradiotherapy for Esophageal Squamous Cell Carcinoma

**DOI:** 10.1155/2022/5635071

**Published:** 2022-06-03

**Authors:** Tae Hoon Lee, Hak Jae Kim, Byoung Hyuck Kim, Chang Hyun Kang, Bhumsuk Keam, Hyeon Jong Moon, Yong Won Seong, Suzy Kim

**Affiliations:** ^1^Department of Radiation Oncology, Seoul National University Hospital, Seoul, Republic of Korea; ^2^Cancer Research Institute, College of Medicine, Seoul National University, Seoul, Republic of Korea; ^3^Institute of Radiation Medicine, Medical Research Center, Seoul National University, Seoul, Republic of Korea; ^4^Department of Radiation Oncology, Seoul Metropolitan Government-Seoul National University Boramae Medical Center, Seoul, Republic of Korea; ^5^Department of Thoracic and Cardiovascular Surgery, Seoul National University Hospital, Seoul, Republic of Korea; ^6^Department of Internal Medicine, Seoul National University Hospital, Seoul, Republic of Korea; ^7^Department of Thoracic and Cardiovascular Surgery, Seoul Metropolitan Government-Seoul National University Boramae Medical Center, Seoul, Republic of Korea

## Abstract

The purpose of this study was to evaluate the feasibility of small primary gross tumor volume (GTV)-to-clinical target volume (CTV) margin expansion in neoadjuvant chemoradiation for esophageal squamous cell carcinoma. Medical records of 139 patients with locally advanced esophageal squamous cell carcinoma who underwent neoadjuvant chemoradiation and radical esophagectomy were retrospectively reviewed. Patients treated with longitudinal primary GTV-to-CTV margin expansion of 2 cm and no additional expansion of the CTV through the esophagus were classified into a small margin (SM) group (37 patients). The remaining 102 patients were classified as a large margin (LM) group. Patterns of recurrence including local and out-field regional recurrence rates were compared between the two groups. Clinical outcomes including rates of local control, regional control, failure-free survival, and overall survival were also compared. More patients in the SM group underwent paclitaxel + carboplatin, Mckeown esophagectomy, and intensity-modulated radiation therapy than in the LM group. With a median follow-up of 25.6 months, there was no significant difference in the crude rate of local recurrence (10.8% vs. 6.9%, *P*=0.694), out-field regional recurrence (27.0% vs. 19.6%, *P*=0.480), or out-field regional recurrence without in-field recurrence (10.8% vs. 12.7%, *P*=0.988) between the two groups. There was no significant difference in failure-free survival (5-year, 34.4% vs. 30.6%, *P*=0.652) or overall survival (44.1% vs. 38.5%, *P*=1.000), either. Esophageal fistula was not reported in the SM group (0.0% vs. 7.9%, *P*=0.176). In conclusion, a radiation field with 2 cm of longitudinal primary GTV-to-CTV was feasible in the neoadjuvant setting for esophageal squamous cell carcinoma treatment.

## 1. Introduction

Trimodality approach including neoadjuvant chemoradiation and surgery has become the standard treatment for locally advanced esophageal cancer, although the treatment outcome of this approach is still unsatisfactory. For example, the CROSS trial, which established neoadjuvant chemoradiation as a standard, reported a 5-year overall survival rate of 47% in the neoadjuvant chemoradiation arm [1]. Furthermore, there are concerns about the toxicities of trimodality approach and its impact on oncologic outcomes [2]. Therefore, optimized treatment is needed for better outcomes of locally advanced esophageal cancer. In this perspective, several debates persist regarding the radiotherapy (RT) component of trimodality approach. Field design is one of the discussion focuses for RT. There is a tendency toward a smaller RT field recently. For instance, many centers have implemented involved-field irradiation rather than extensive field including elective supraclavicular fossa or celiac axis nodal irradiation. Although an extensive RT field may decrease recurrences in those nodal areas, the effect of elective field to final treatment outcomes including survival rate is not conclusive [3].

Another important point of debate for RT field design is gross tumor volume (GTV)-to-clinical target volume (CTV) margin expansion for primary esophageal lesion. Traditionally, a 5 cm margin above and below the GTV was recommended to cover subclinical disease [4]. However, a recently published guideline suggested a 3 cm margin for GTV-to-CTV expansion based on pathologic examination of esophagectomy specimens [5, 6]. A previous clinical study also suggested that a 2 cm margin for longitudinal GTV-to-CTV expansion was adequate, showing an acceptable locoregional recurrence rate [7]. As these tendencies toward the smaller field continue, concerns about the safety of these field designs also persist. The purpose of this study was to evaluate the feasibility of small longitudinal primary GTV-to-CTV margin expansion in neoadjuvant chemoradiation for esophageal cancer by comparing patterns of recurrence and oncologic outcomes.

## 2. Materials and Methods

### 2.1. Study Population

This study was approved by the institutional review boards of Seoul Metropolitan Government-Seoul National University Boramae Medical Center (IRB no. 30-2021-49) and Seoul National University Hospital (IRB no. H-2105-156-1221) before collecting patient information. Medical records of the patients who underwent neoadjuvant chemoradiation and surgery for locally advanced (T3-4 or N+) esophageal squamous cell carcinoma in two institutions (Seoul Metropolitan Government-Seoul National University Boramae Medical Center and Seoul National University Hospital) from January 2005 to December 2018 were retrospectively reviewed. A total of 188 patients underwent neoadjuvant chemoradiation for esophageal cancer during this period. Seven patients who did not have squamous cell carcinoma histology and nine patients with previous malignancy history in 5 years or concomitant malignancy were excluded. Seven patients who were irradiated less than 40 Gy were also excluded. Among the remaining 165 patients, 26 patients could not undergo radical esophagectomy. As a result, 139 patients were included in the analysis.

### 2.2. Treatment and Definition of the Groups

Patients underwent simulation computerized tomography (CT) scan in the supine position with both arms abducted and immobilized with wing boards. The primary GTV was defined as an esophageal tumor visualized on CT, positron emission tomography (PET), and endoscopy. Primary CTV was generated with 2.0 to 5.0 cm longitudinal and a 0.5 to 1.0 cm radial margin expansion. If suspected metastatic lymph nodes were confirmed on staging work-up and visible on simulation CT, they were delineated as nodal GTV. Nodal CTV was generated with 0.5 to 1.0 cm margin expansion in all directions. The planning target volume (PTV) was generated by applying 0.5 to 1.0 cm margin around CTVs. Before 2014, RT often consisted of two courses, and reduced-field RT was followed immediately after the first course. In reduced-field RT, primary GTV-to-CTV margin expansion was 0 to 2.0 cm for a longitudinal direction and 0 to 1 cm for a radial direction. The PTV for reduced-field RT was defined as CTV for reduced-field RT with 0 to 1.0 cm margin expansion. Elective RT field in a supraclavicular or celiac axis lymph node area was decided by the treating radiation oncologist. Both three-dimensional conformal radiation therapy (3D-CRT) and intensity-modulated radiation therapy (IMRT) were used. Chemotherapy was administered concurrently with RT, and the regimen was selected by the treating medical oncologist.

After completing chemoradiation, patients underwent radical esophagectomy. Transthoracic esophagectomy was preferred, but the exact surgical method was at the discretion of the treating thoracic surgeon. Adjuvant chemotherapy was administered to the patients with an advanced surgical stage. The patients with positive surgical margin or gross residual disease underwent postoperative RT.

**Figure 1:**
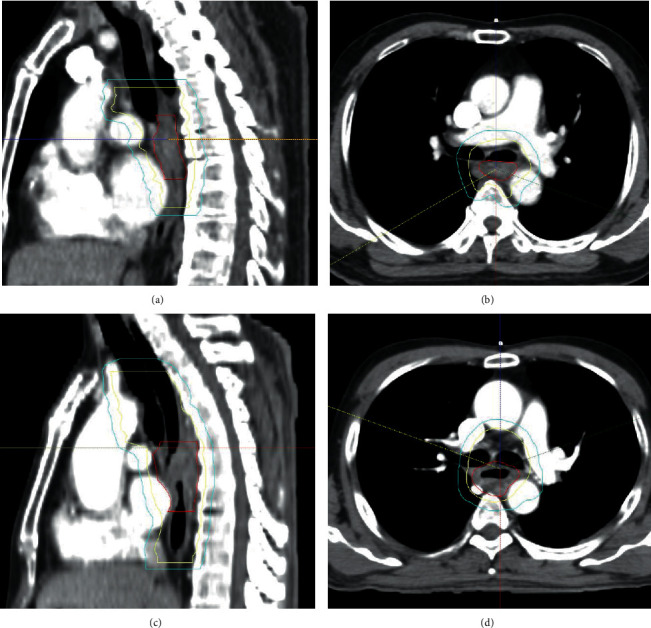
Examples of target delineation of small margin (SM) and large margin (LM) groups. Red, yellow, and cyan lines indicate gross tumor volume (GTV), clinical target volume (CTV), and planning target volume (PTV), respectively. (a) Sagittal and (b) axial cuts from the representative case of the SM group illustrating target delineation with longitudinal primary GTV-to-CTV of 2 cm and no additional elective field for the longitudinal direction. (c) Sagittal and (d) axial cuts of representative case from the LM group illustrating target delineation with more extensive CTV, especially in the longitudinal direction.

Patients with longitudinal primary GTV-to-CTV margin expansion of 2 cm and no additional longitudinal expansion of the CTV by elective coverage of mediastinum through esophagus beyond initial primary GTV-to-CTV expansion were classified as a small margin (SM) group. Coverage of esophagus within the same axial plane with involved nodal CTV was allowed. Elective irradiation of supraclavicular or celiac axis area was also permitted. As a result, 37 (26.6%) patients were included in the SM group. The remaining 102 patients were classified as a large margin (LM) group. Examples of target delineation of SM group and LM group are illustrated in Figure 1.

### 2.3. Patterns of Recurrence and Clinical Outcomes

Exact sites of disease recurrence occurring within the follow-up period were categorized into local recurrence, regional recurrence, and distant metastasis. Regional recurrences were further categorized into in-field and out-field recurrences. Disease in paraesophageal and celiac axis lymph node was considered as regional spread, while disease in supraclavicular fossa was considered as distant metastasis, as described in AJCC/UICC staging 8th edition [8, 9]. Crude rates of local recurrence, in-field /out-field regional recurrence, and distant metastasis were compared between the SM and LM groups by chi-square test. Locations of distant metastasis were also compared between two groups by chi-square test.

Rates of local control (LC), regional control (RC), failure-free survival (FFS), and overall survival (OS) were calculated using the Kaplan–Meier method. An LC event was defined as recurrence of disease in the anastomotic site and an RC event was defined as recurrence of disease in the mediastinal and celiac axis lymph node area. An FFS event was defined as any failure or death, while an OS event was defined as the death of a patient from any cause. Survival data were retrieved from the resident registration system of the government of the Republic of Korea. LC, RC, FFS, and OS of two groups were compared by log-rank test. Univariate analysis was performed for LC, RC, FFS, and OS to identify potential preoperative prognostic factors affecting treatment outcomes. Statistically significant or marginally significant variables (*P* < 0.1) and RT field (SM vs. LM group) were incorporated into the multivariate analysis using the Cox proportional hazards model to investigate the effect of RT field and other potential variables on the clinical outcomes. Rates of major toxicities including esophageal stricture requiring intervention and fistula were calculated and compared by chi-square test between the two groups. *P* value less than 0.05 was defined as statistically significant throughout all statistical tests. All statistical analyses were performed using R 4.1.1 (The R Foundation for Statistical Computing, Vienna, Austria).

## 3. Results

### 3.1. Patient Characteristics

Patient characteristics are summarized in Table 1. A median follow-up period was 25.6 months (range, 3.0 to 141.5 months) for all patients and 58.3 months (range, 19.5 to 129.7 months) for surviving patients only. Total cumulative prescribed radiation dose had a median of 45 Gy (range, 40 to 54 Gy). Dose per fraction was 1.8 Gy for most (95%) patients. Six (4.3%) patients had dose per fraction of 2 Gy. One (0.7%) patient had 2.25 Gy per fraction. Reduced-field RT was conducted in 44 (31.6%) patients. The prescribed dose of reduced-field RT was 5.4 Gy (range, 3.6 to 9.0 Gy) except for three patients. Total cumulative RT dose was lower in the SM group than in the LM group due to less usage of reduced-field irradiation. All but one patient who was irradiated less than 50.4 Gy did not have a reduced-field irradiation, while patients irradiated ≥50.4 Gy had reduced-field irradiation. 3D-CRT was applied for RT planning in 100 (71.9%) patients. The remaining 39 (28.1%) patients used IMRT. Thirty (29.1%) patients underwent supraclavicular elective irradiation. No patient received elective irradiation in the celiac axis lymph node area. In the LM group, a median actual longitudinal distance between the primary GTV and the superior margin of the CTV covering the esophagus was 5.6 cm (range, 2.0 to 16.2 cm), and a median distance between the primary GTV and the inferior margin of the CTV was 2.0 cm (range, 2.0 to 7.8 cm). The distance between the primary GTV and the superior margin of the CTV was longer because upper mediastinal elective CTV was frequently set by the treating radiation oncologist, while GTV-to-CTV expansion to an inferior direction was often limited by the gastroesophageal junction.

Regarding concurrent chemotherapy, weekly paclitaxel + carboplatin was applied to 76 (54.7%) patients and 5-fluorouracil (5-FU) + cisplatin was applied to 51 (36.7%) patients. Weekly cisplatin was administered to 10 (7.2%) patients. Cetuximab + paclitaxel + carboplatin and docetaxel + cisplatin were used in one (0.7%) patient each. There was a significantly increased use of weekly paclitaxel + carboplatin after 2014 (before 2014, 21.3% vs. after 2014, 80.8%, *P* < 0.001). Planned chemotherapy was completely administered to 126 (90.6%) patients.

Almost all (99.3%) of patients underwent transthoracic esophagectomy, and one (0.7%) patient underwent transhiatal esophagectomy. Among patients who underwent transthoracic esophagectomy, Mckeown esophagectomy was applied to 81 (58.3%) patients, and Ivor–Lewis esophagectomy was applied to 57 (41.0%) patients. Regarding lymph node dissection, 77 (55.4%) patients underwent a 3-field dissection and 58 (41.7%) patients underwent a 2-field dissection. Four (2.9%) patients had no information about the type of lymph node dissection. Median interval between the end of chemoradiation and surgery was 42 days (range, 23 to 95 days). Adjuvant chemotherapy was administered to 24 (17.3%) patients. The regimen of adjuvant chemotherapy was 5-FU + cisplatin for 20 patients and docetaxel + cisplatin for three patients. One patient went to another institution for adjuvant chemotherapy with an unknown regimen. Five (3.6%) patients with R1 resection underwent postoperative RT to the esophageal tumor bed, and the median dose was 16.2 Gy (range, 14.4 to 20.0 Gy).

There were some differences in treatment between the SM and LM group mainly due to changes in dominant treatment method by period. In the SM group, 21 (56.8%) patients started chemoradiation in 2017 and 2018, while in the LM group, 20 (19.6%) patients started chemoradiation in the same period. More patients in the SM group underwent paclitaxel + carboplatin as a chemotherapeutic regimen (78.4% vs. 46.1%), IMRT for RT planning (43.2% vs. 22.5%), Mckeown esophagectomy (83.8% vs. 49.0%), and 3-field lymph node dissection (86.5% vs. 44.1%) than in the LM group. Median number of dissected lymph nodes was 47 (range, 5 to 113) in the entire cohort. No increase in R1 resection was observed in the SM group (2.7% vs. 7.8%).

In addition, the SM group had more N2/3 disease (37.8% vs. 17.6%). Patients in the SM group had more upper esophageal (above azygos vein) involvement (48.6% vs. 17.6%) but less lower esophageal (below inferior pulmonary vein) involvement (35.1% vs. 56.9%). This was due to a tendency to extend the CTV to the upper mediastinal lymph node area in the nonupper esophageal primary lesion, resulting in an inclusion of part of upper esophagus in the CTV, which made the patient ineligible to be classified into the SM group.

Twenty-nine (28.4%) patients with clinical M1 disease were included in this study. Twenty-two patients (8 from the SM group and 14 from the LM group) had distant metastasis in the supraclavicular lymph node only and 2 patients (one from the SM group and one from the LM group) had abdominal para-aortic lymph node metastasis. Four patients (one from the SM group and 3 from the LM group) had neck lymph node metastasis at the time of diagnosis. One patient in the LM group had lung metastasis, which was histologically confirmed before chemoradiation. The lung tumor was regressed during chemoradiation. The patient underwent metastasectomy and radical esophagectomy.

### 3.2. Patterns of Recurrence

Patterns of recurrence occurring during the follow-up period are summarized in Table 2. No significant difference between the two groups was observed for each failure site. There was no difference in the crude rate of local recurrence (10.8% vs. 6.9%, *P*=0.684), all out-field regional recurrence (27.0% vs. 19.6%, *P*=0.480), or crude rate of isolated out-field regional recurrence without in-field recurrence (10.8% vs. 12.7%, *P*=0.988) between SM and LM groups. The most frequent site of distant metastasis was lung. Forty (28.8%) patients had lung metastasis during the follow-up period. Metastases to nonregional lymph nodes (27 patients, 19.4%) and liver (20 patients, 14.4%) were also frequent. Sites of distant metastasis showed no difference between the two groups. One (2.7%) patient in the SM group and four (3.9%) patients in the LM group had celiac axis lymph node failure (*P*=1.000). There was no significant difference in the crude rate of supraclavicular lymph node failure between the two groups (8.1% vs. 15.7%, *P*=0.384).

### 3.3. Clinical Outcomes

**Figure 2:**
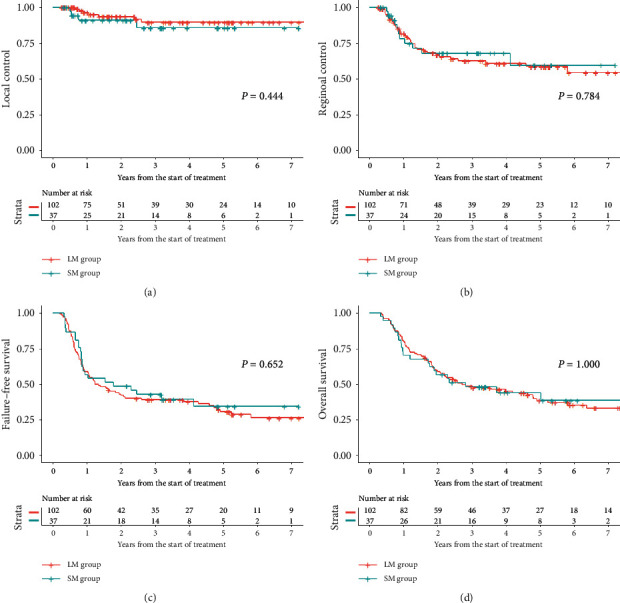
Kaplan–Meier curves of (a) local control, (b) regional control, (c) failure-free survival, and (d) overall survival.

The Kaplan–Meier curves of LC, RC, FFS, and OS are illustrated in Figure 2. Three-year and 5-year LC rates were 85.7% and 85.7% in the SM group and 89.6% and 89.6% in the LM group, respectively. Three-year and 5-year RC rates were 68.1% and 59.6% in the SM group and 62.5% and 58.7% in the LM group, respectively. There were no significant differences in LC (*P*=0.444) or RC (*P*=0.784) rates between the two groups. Three-year and 5-year FFS rates were 42.9% and 34.4% in the SM group and 39.0% and 30.6% in the LM group, respectively. Three-year and 5-year OS rates were 48.1% and 44.1% in the SM group and 48.6% and 38.5% in the LM group, respectively. There was no significant difference in FFS (*P*=0.652) or OS (*P*=1.000) between the two groups.

Results of univariate and multivariate analyses for clinical outcomes are summarized in Supplementary Table 1 and Table 3, respectively. Completeness of chemotherapy was associated with better LC in univariate analysis. In a multivariate model with RT field (SM vs. LM group), this significant association was maintained, while field size was not associated with LC. No variables showed association with RC. Age, supraclavicular elective irradiation, and longitudinal length of GTV were associated with FFS in univariate analysis. In multivariate analysis, older age and longer longitudinal length of GTV were associated with worse FFS. Age, upper thoracic involvement, supraclavicular elective irradiation, and longitudinal length of GTV were associated with OS in univariate analysis. In multivariate analysis, older age and longer longitudinal length of GTV were associated with worse OS. RT field was not associated with FFS or OS in multivariate models.

Regarding major toxicities, four (10.8%) patients from the SM group and 19 (18.8%) patients from the LM group had esophageal stricture requiring intervention. Eight (7.9%) patients from the LM group had esophageal fistula, although no patient from the SM group had such event. Overall, four (10.8%) patients from the SM group and 24 (23.8%) patients from the LM group had either stricture or fistula. These toxicity rates were not significantly different between the two groups (stricture, *P*=0.390; fistula, *P*=0.176; stricture or fistula, *P*=0.151).

## 4. Discussion

The current study investigated the effect of using the RT field of small or large primary GTV-to-CTV margin expansion on the failure patterns and clinical outcomes in neoadjuvant chemoradiotherapy for esophageal squamous cell carcinoma and showed that small longitudinal primary GTV-to-CTV margin expansion did not significantly harm the treatment outcomes of esophageal squamous cell carcinoma.

Implementing a small RT field in our group was a result of multidisciplinary discussion, especially between thoracic surgeons and radiation oncologists. Several studies have shown that a higher number of lymph node dissected resulted in better treatment outcomes [10, 11], thus favoring extensive lymph node dissection, although this concept is challenged by reports published after the implementation of neoadjuvant chemoradiation [12]. Even after publications of randomized evidence, several groups of surgeons mainly from East Asia have emphasized the importance of extensive lymph node dissection [13, 14]. As easily assumed from the median number of removed lymph nodes in this cohort, which is close to 50, thoracic surgeons in our institutions also support extensive lymphadenectomy. Major concerns of these surgeons about neoadjuvant treatment are technical difficulties of surgical approach to the mediastinum due to fibrosis and adhesion caused by radiation, which might impact postoperative morbidities and mortalities [15, 16]. Thoracic surgeons in our institution constantly suggested to move toward smaller RT fields. As reports about the feasibility of involved-field irradiation for esophageal squamous cell carcinoma of Asian population are accumulated [17, 18] and smaller longitudinal primary GTV-to-CTV margin expansion than the traditional RT field was applied, radiation oncologists of our institutions also start to favor smaller RT fields.

Even with the trend toward smaller fields, 2 cm of longitudinal primary GTV-to-CTV margin is smaller than the lower limit of generally accepted margin expansion. Many radiation oncologists are reluctant to reduce longitudinal primary GTV-to-CTV margin to be smaller than 3 cm based on pathological and clinical data [6, 7]. Furthermore, there is a report that the residual tumor after neoadjuvant chemoradiation might have a devastating effect on survival rate [19]. However, the clinical outcomes of SM group were not inferior to that of LM group in the current study, which included East Asian esophageal squamous cell carcinoma patients with a relatively advanced clinical stage. Recently, our group has implemented involved-field irradiation for neoadjuvant chemoradiation for esophageal cancer. We are waiting for maturation of patient cohort with a small primary GTV-to-CTV margin and strict involved-field irradiation, which does not have additional RT field outside of initially generated primary and nodal CTV by margin expansion, for further reduction of RT field.

The current study reported that 33.1% of all patients experienced regional recurrence. This rate is relatively higher than the locoregional recurrence rate of around 20% from the prospective series that applied neoadjuvant chemoradiation [1, 20–22]. When interpreting recurrence patterns of esophageal cancer, the histological difference needs to be considered. It is acknowledged that squamous cell carcinoma has a tendency to have more locoregional recurrence, while adenocarcinoma has a tendency to have more distant metastasis [23, 24]. The patient cohort of the current study consisted of squamous cell carcinoma only, which is dominant in the East Asian population. Baseline characteristics of the patients also should be taken into account. Only 15 patients (10.8%) of the cohort had diseases confined to the esophagus, and a high percentage of the patients had regional and nonregional lymph node metastasis. Furthermore, the 7th edition AJCC/UICC system defines any paraesophageal lymph nodes from cervical nodes to celiac nodes as regional lymph nodes regardless of location of primary lesion within esophagus, which is broader than the definition from the previous edition [25]. Some prior reports used the 6th edition of AJCC/UICC system. The rate of regional recurrence might be higher in the current study even with a similar recurrence pattern when compared with these reports.

In this current study, major toxicities including esophageal stricture that required intervention and fistula were reported. Being a retrospective study, minor toxicities were not well-documented and thus were not included in the analysis. Reported rates of major toxicities were lower in the SM group and no patient in the SM group had esophageal fistula, although the differences of these rates did not reach statistical significance. It is well known that the RT field may impact toxicity rates [16, 18]. Smaller RT field might be beneficial to lower surgical morbidities. This should be addressed in further studies with a larger cohort.

It is hard to conclude the impact of different chemotherapeutic regimens of chemoradiotherapy on the clinical outcomes. Conflictive results of comparison studies between paclitaxel + carboplatin and 5-FU + cisplatin have been reported [26, 27]. In the current study, no difference in treatment outcomes by chemotherapeutic regimen was observed, but incomplete chemotherapy was associated with lower LC. Additional research on optimal combination of chemotherapy regimens is needed in the future.

Statistically significant prognostic factors for FFS and OS in the current study were age and longitudinal length of primary GTV. The length of primary lesion is a well-known risk factor for survival [28]. We used the length of primary GTV instead due to the lack of endoscopic description for the length of esophageal lesion. Our result was concordant with previous studies using the length measured from staging work-ups. Other differences in treatment such as RT technique (3D-CRT vs. IMRT) and type of surgical approach did not lead to significant differences in the treatment outcomes. This might be due to the lack of statistical power, and further studies are needed to clarify this.

This study has several limitations. First, the effect of RT field was hard to isolate due to the retrospective nature of this study. Several differences of patient characteristics and treatment factors were observed between the two groups, although univariate and multivariate analyses did not show any evidence of worse clinical outcomes for the SM group. Further studies with more comparable or prospective cohorts would be warranted. Second, principles for target volume delineation were gradually changed, and this might have mitigated potential differences in outcomes between the two groups. Third, the patient selection factor should be considered. Additional RT field in the mediastinum was applied in discretion of the treating radiation oncologist and patients with a high risk of mediastinal lymph node metastasis were most likely to be implemented additional mediastinal RT field encompassing both lymph node area and esophagus, which made the patient ineligible for being classified into the SM group. Nevertheless, the current study showed that limited primary GTV-to-CTV margin expansion resulted in comparable clinical outcomes and patterns of failure, contrary to concerns from some radiation oncologists.

In conclusion, 2 cm of longitudinal primary GTV-to-CTV margin expansion is feasible for neoadjuvant chemoradiation for locally advanced esophageal squamous cell carcinoma. Although not reaching statistical significance, no patient in the SM group had an esophageal fistula. Caution would be needed when applying this principle as target volume delineation should be tailored by each institution with interdepartmental discussion. A further study applying both small margin and involved-field irradiation is underway.

## Figures and Tables

**Table 1:** Patient characteristics.

Characteristics	Small margin group (*N* = 37)	Large margin group (*N* = 102)	*P* value
Age (years, median, range)	61.5 (39.2–76.7)	62.4 (35.2–81.6)	0.567
Sex			0.760
Male	34 (91.9%)	97 (95.1%)	
Female	3 (8.1%)	5 (4.9%)	
ECOG performance status			0.625
0	8 (21.6%)	15 (14.7%)	
1	28 (75.7%)	84 (82.4%)	
2	1 (2.7%)	3 (2.9%)	
Differentiation (prechemoradiation)			0.795^*∗*^
Well differentiated	2 (5.4%)	9 (8.8%)	
Moderately differentiated	27 (73.0%)	73 (71.6%)	
Poorly differentiated	3 (8.1%)	10 (9.8%)	
Unknown	5 (13.5%)	10 (9.8%)	
Subsite			0.008
Upper thoracic	13 (35.1%)	13 (12.7%)	
Upper and middle thoracic	5 (13.5%)	5 (4.9%)	
Middle thoracic	6 (16.2%)	26 (25.5%)	
Middle and lower thoracic	2 (5.4%)	11 (10.8%)	
Lower thoracic	11 (29.7%)	47 (46.1%)	
Clinical T stage			0.801^*∗*^
cT1	2 (5.4%)	4 (3.9%)	
cT2	7 (18.9%)	26 (25.5%)	
cT3	27 (73.0%)	66 (64.7%)	
cT4	1 (2.7%)	4 (3.9%)	
Unknown	0 (0.0%)	2 (2.0%)	
Clinical N stage			0.012
cN0	7 (18.9%)	16 (15.7%)	
cN1	16 (43.2%)	68 (66.7%)	
cN2	12 (32.4%)	18 (17.6%)	
cN3	2 (5.4%)	0 (0.0%)	
Clinical M stage			0.400
cM0	27 (73.0%)	83 (81.4%)	
cM1	10 (27.0%)	19 (18.6%)	
Chemotherapy regimen			0.003
5-FU + cisplatin	6 (16.2%)	45 (44.1%)	
Paclitaxel + carboplatin	29 (78.4%)	47 (46.1%)	
Others	2 (5.4%)	10 (9.8%)	
Chemotherapy completed			0.527
Yes	35 (94.6%)	91 (89.2%)	
No	2 (5.4%)	11 (10.8%)	
Radiotherapy technique			0.029
3D-CRT	21 (56.8%)	79 (77.5%)	
IMRT	16 (43.2%)	23 (22.5%)	
Total radiation dose			0.001
<50.4 Gy	34 (91.9%)	62 (60.8%)	
≥50.4 Gy	3 (8.1%)	40 (39.2%)	
Supraclavicular elective irradiation			1.000
Yes	8 (21.6%)	22 (21.6%)	
No	29 (78.4%)	80 (78.4%)	
Longitudinal length of primary GTV (cm, mean ± SD)	5.8 ± 1.9	6.5 ± 3.1	0.112
CTV (cm^3^, mean ± SD)	179.4 ± 67.0	222.6 ± 81.5	0.005
PTV (cm^3^, mean ± SD)	419.0 ± 120.6	501.3 ± 150.4	0.003
Type of surgery			0.001
Mckeown	31 (83.8%)	50 (49.0%)	
Ivor–Lewis	6 (16.2%)	51 (50.0%)	
Transhiatal	0 (0.0%)	1 (1.0%)	
Lymph node dissection			<0.001^*∗*^
2-field	5 (13.5%)	53 (52.0%)	
3-field	32 (86.5%)	45 (44.1%)	
Unknown	0 (0.0%)	4 (3.9%)	
Number of lymph nodes harvested (median, range)	53 (16–93)	45 (5–113)	0.218
Margin status			0.527
R0	36 (97.3%)	94 (92.2%)	
R1	1 (2.7%)	8 (7.8%)	
Pathologic complete resolution			0.880
Yes	9 (24.3%)	28 (27.5%)	
No	28 (75.7%)	74 (72.5%)	
Postoperative radiotherapy			0.392
Yes	0 (0.0%)	5 (4.9%)	
No	37 (100.0%)	97 (95.1%)	
Adjuvant chemotherapy			0.955
Yes	7 (18.9%)	17 (16.7%)	
No	30 (81.1%)	85 (83.3%)	

^*∗*^The patients with unknown value were excluded from the calculation of this *P* value. Abbreviations: ECOG, Eastern Cooperative Oncology Group; 5-FU, 5-fluorouracil; 3D-CRT, 3-dimensional conformal radiation therapy; IMRT, intensity-modulated radiation therapy; GTV, gross tumor volume; CTV, clinical target volume; PTV, planning target volume.

**Table 2:** Patterns of recurrence.

Site of recurrence	Small margin group (*N* = 37)	Large margin group (*N* = 102)	*P* value
Any recurrence	18 (48.6%)	56 (54.9%)	0.645
Local recurrence	4 (10.8%)	7 (6.9%)	0.684
Regional recurrence	11 (29.7%)	35 (34.3%)	0.761
In-field recurrence	7 (18.9%)	22 (21.6%)	0.917
In-field recurrence without out-field recurrence	1 (2.7%)	15 (14.7%)	0.097
Out-field recurrence	10 (27.0%)	20 (19.6%)	0.480
Out-field recurrence without in-field recurrence	4 (10.8%)	13 (12.7%)	0.988
In-field and out-field recurrences	6 (16.2%)	7 (6.9%)	0.179
Distant metastasis	16 (43.2%)	51 (50.0%)	0.608
Lung	10 (27.0%)	30 (29.4%)	0.950
Nonregional lymph node	5 (13.5%)	22 (21.6%)	0.413
Supraclavicular fossa	3 (8.1%)	16 (15.7%)	0.384
Neck	2 (5.4%)	9 (8.8%)	0.761
Intra-abdominal	2 (5.4%)	9 (8.8%)	0.761
Axilla	2 (5.4%)	1 (1.0%)	0.354
Liver	4 (10.8%)	16 (15.7%)	0.652
Bone	2 (5.4%)	12 (11.8%)	0.434
Pleural seeding	2 (5.4%)	8 (7.8%)	0.904
Others	5^†^ (13.5%)	13^‡^ (12.7%)	1.000

^†^Adrenal gland = 2, kidney = 2, and hypopharynx = 1. ^‡^Peritoneal seeding = 5, adrenal gland = 3, kidney = 3, pancreas = 2, hypopharynx = 1, brain = 1, cecum = 1, and psoas muscle = 1. One patient had both peritoneal seeding and pancreatic metastasis, and another patient had both adrenal and cecal metastasis. One patient had peritoneal, renal, and psoas muscle metastasis.

**Table 3:** Multivariate analysis of clinical outcomes.

Characteristics (comparison vs. reference)	Local control			Failure-free survival			Overall survival		
	HR	95% CI	*P* value	HR	95% CI	*P* value	HR	95% CI	*P* value
Age (continuous)	—	—	—	1.033	1.006–1.061	0.018	1.041	1.011–1.072	0.007
Upper thoracic involvement (yes vs. no)	—	—	—	—	—	—	0.759	0.398–1.445	0.401
Supraclavicular elective irradiation (yes vs. no)	—	—	—	0.671	0.382–1.180	0.166	0.798	0.399–1.593	0.522
Longitudinal length of primary GTV (continuous)	—	—	—	1.106	1.031–1.186	0.005	1.093	1.017–1.174	0.016
Chemotherapy completed (yes vs. no)	0.160	0.046–0.563	0.004	—	—	—	—	—	—
Field size (small margin vs. large margin group)	1.997	0.571–6.980	0.279	1.057	0.650–1.718	0.824	1.268	0.745–2.157	0.382

Abbreviations: HR, hazard ratio; CI, confidence interval; GTV, gross tumor volume.

## Data Availability

Research data are stored in an institutional repository and will be shared upon request to the corresponding author.

## Supplementary Materials

Supplementary Materials Supplementary Table 1. Univariate analysis of clinical outcomes.
