# Supplementary material for: Comparable Clinical Outcome Using Small or Large Gross Tumor Volume-to-Clinical Target Volume Margin Expansion in Neoadjuvant Chemoradiotherapy for Esophageal Squamous Cell Carcinoma
Source: J Oncol. 2022 Jun 3;2022:5635071. doi: 10.1155/2022/5635071 (PMC9187480; doi:10.1155/2022/5635071)
Supplement: Supplementary Materials — Supplementary Table 1. Univariate analysis of clinical outcomes. [file 5635071.f1.docx]

**Supplementary Table 1. Univariate analysis of clinical outcomes**

| **Characteristics**  **(comparison vs reference)** | **Local control** | | | **Regional control** | | | | **Failure-free survival** | | | | **Overall survival** | | | |
| --- | --- | --- | --- | --- | --- | --- | --- | --- | --- | --- | --- | --- | --- | --- | --- |
|  | HR | 95% CI | *P*-value | | HR | 95% CI | *P*-value | | HR | 95% CI | *P*-value | | HR | 95% CI | *P*-value |
| Age (continuous) | 1.053 | 0.972–1.141 | 0.208 | | 1.023 | 0.986–1.060 | 0.230 | | 1.027 | 1.002–1.054 | 0.037 | | 1.038 | 1.009–1.067 | 0.009 |
| Sex (female vs male) | - | - | -* | | 1.033 | 0.320–3.331 | 0.957 | | 0.648 | 0.238–1.764 | 0.396 | | 0.588 | 0.186–1.862 | 0.367 |
| ECOG performance status (>2 vs ≤1) | - | - | -* | | 2.351 | 0.727–7.600 | 0.153 | | 1.739 | 0.638–4.746 | 0.280 | | 1.506 | 0.475–4.774 | 0.487 |
| Differentiation (poorly differentiated vs well & moderately) | 0.826 | 0.103–6.615 | 0.857 | | 0.683 | 0.242–1.925 | 0.470 | | 0.561 | 0.257–1.221 | 0.145 | | 0.640 | 0.293–1.395 | 0.261 |
| Upper thoracic involvement (yes vs no) | 0.968 | 0.257–3.654 | 0.962 | | 0.691 | 0.342–1.396 | 0.303 | | 0.821 | 0.508–1.326 | 0.419 | | 0.633 | 0.372–1.079 | 0.093 |
| Middle thoracic involvement (yes vs no) | 0.348 | 0.075–1.611 | 0.177 | | 0.866 | 0.476–1.576 | 0.637 | | 1.104 | 0.731–1.668 | 0.637 | | 1.060 | 0.687–1.635 | 0.794 |
| Lower thoracic involvement (yes vs no) | 1.864 | 0.545–6.370 | 0.321 | | 1.334 | 0.746–2.386 | 0.331 | | 1.089 | 0.725–1.637 | 0.681 | | 1.348 | 0.879–2.067 | 0.172 |
| T stage (T3 & 4 vs T1 & 2) | 3.901 | 0.494–30.83 | 0.197 | | 1.490 | 0.737–3.012 | 0.267 | | 1.457 | 0.893–2.378 | 0.132 | | 1.348 | 0.813–2.233 | 0.247 |
| N stage (N+ vs N0) | 0.508 | 0.109–2.361 | 0.388 | | 1.250 | 0.447–3.498 | 0.671 | | 1.431 | 0.661–3.094 | 0.363 | | 2.002 | 0.804–4.986 | 0.136 |
| M stage (M1 vs M0) | 1.400 | 0.371–5.283 | 0.620 | | 1.190 | 0.604–2.344 | 0.615 | | 1.193 | 0.733–1.940 | 0.477 | | 1.075 | 0.638–1.812 | 0.785 |
| Field size (small margin vs large margin group) | 1.609 | 0.471–5.499 | 0.448 | | 0.909 | 0.461–1.792 | 0.783 | | 0.898 | 0.560–1.439 | 0.655 | | 1.000 | 0.610–1.640 | 1.000 |
| RT technique (IMRT vs 3D-CRT) | 1.111 | 0.295–4.192 | 0.876 | | 1.341 | 0.703–2.561 | 0.373 | | 1.270 | 0.803–2.007 | 0.306 | | 1.241 | 0.758–2.030 | 0.391 |
| Total dose (≥50.4 Gy vs <50.4 Gy) | 2.102 | 0.640–6.904 | 0.221 | | 1.074 | 0.578–1.997 | 0.820 | | 1.410 | 0.925–2.148 | 0.110 | | 1.391 | 0.898–2.155 | 0.139 |
| Supraclavicular elective irradiation (yes vs no) | 1.843 | 0.538–6.310 | 0.330 | | 0.740 | 0.356–1.539 | 0.420 | | 0.633 | 0.369–1.089 | 0.098 | | 0.553 | 0.306–1.002 | 0.051 |
| Longitudinal length of primary GTV (continuous) | 1.025 | 0.820–1.282 | 0.827 | | 1.068 | 0.967–1.181 | 0.193 | | 1.090 | 1.014–1.172 | 0.019 | | 1.079 | 1.003–1.161 | 0.041 |
| CTV (continuous) | 1.000 | 0.992–1.009 | 0.907 | | 1.002 | 0.999–1.006 | 0.218 | | 1.002 | 1.000–1.005 | 0.109 | | 1.002 | 0.999–1.005 | 0.114 |
| PTV (continuous) | 0.999 | 0.994–1.003 | 0.530 | | 1.000 | 0.999–1.002 | 0.810 | | 1.001 | 1.000–1.003 | 0.122 | | 1.001 | 1.000–1.003 | 0.138 |
| Chemotherapy regimen (vs FP) |  |  |  | |  |  |  | |  |  |  | |  |  |  |
| TC | 0.734 | 0.196–2.747 | 0.647 | | 0.625 | 0.333–1.174 | 0.144 | | 0.718 | 0.466–1.107 | 0.134 | | 0.733 | 0.465–1.155 | 0.181 |
| Others | 2.109 | 0.386–11.52 | 0.389 | | 1.595 | 0.669–3.802 | 0.292 | | 1.419 | 0.707–2.848 | 0.325 | | 1.359 | 0.672–2.748 | 0.394 |
| Chemotherapy completed (yes vs no) | 0.179 | 0.052–0.615 | 0.006 | | 0.777 | 0.329–1.833 | 0.564 | | 1.085 | 0.545–2.158 | 0.817 | | 0.834 | 0.428–1.626 | 0.595 |
| Type of esophagectomy (Mckeown vs Ivor-Lewis) | 0.934 | 0.285–3.063 | 0.911 | | 0.870 | 0.487–1.556 | 0.639 | | 1.018 | 0.673–1.541 | 0.932 | | 1.067 | 0.690–1.651 | 0.770 |
| Lymph node dissection (3-field vs 2-field) | 0.779 | 0.226–2.693 | 0.693 | | 0.971 | 0.536–1.760 | 0.924 | | 1.015 | 0.669–1.540 | 0.943 | | 1.059 | 0.684–1.639 | 0.798 |

* Cannot be calculated as no event was occurred in certain group.

Abbreviations: HR, hazard ratio; CI, confidence interval; ECOG, Eastern Cooperative Oncology Group; GTV, gross tumor volume; CTV, clinical target volume; PTV, planning target volume; FP, 5-FU + cisplatin; TC, paclitaxel + carboplatin.
